# Pharmacological and Pharmacokinetic Profiling of *Asphodelus microcarpus* Phytochemicals: *UHPLC–MS/MS* Characterization, Molecular Docking, and ADMET Analysis

**DOI:** 10.1002/fsn3.72096

**Published:** 2026-07-11

**Authors:** Mohammed Roubi, Youness Mahdi, Ayoub Farihi, Chahrazad Belkhiri, Mohammed Dalli, Salah‐Eddine Azizi, Nour Elhouda Daoudi, Raffaele Conte, Mohammed Hawwal, Mohammed Alzahrani, Omer M. Almarfadi, Nadia Gseyra, Ramzi A. Mothana

**Affiliations:** ^1^ Laboratory of Bioresources, Biotechnology, Ethnopharmacology and Health, Faculty of Sciences, Mohammed First University Oujda Morocco; ^2^ Laboratory for Agricultural Production Improvement, Biotechnology and Environment, Mohammed First University Oujda Morocco; ^3^ Higher Institute of Nursing Professions and Health Techniques Oujda Morocco; ^4^ Research Institute on Terrestrial Ecosystems (IRET)‐CNR 80131 Naples Italy; ^5^ University of Naples ‘Federico II’ Naples Italy; ^6^ Department of Pharmacognosy College of Pharmacy, King Saud University Riyadh Saudi Arabia

**Keywords:** antidiabetic, antioxidant, *Asphodelus microcarpus*, *UHPLC–MS/MS*

## Abstract

This study investigates the phytochemical composition and biological activities of *Asphodelus microcarpus*, a plant traditionally utilized in Moroccan medicine. Employing advanced *UHPLC–MS/MS* analysis, both ethanolic and methanolic extracts of *A. microcarpus* flowers were thoroughly characterized. The analysis revealed the presence of 16 compounds in the methanolic extract and 15 in the ethanolic extract, with isorhamnetin and luteolin identified as major constituents. Notably, the antioxidant gamma‐tocopherol was also detected. Further in vitro evaluations demonstrated significant antioxidant and antidiabetic potentials. To elucidate the underlying mechanisms, molecular docking simulations were performed. These simulations indicated strong binding affinities of key compounds, such as isorhamnetin and luteolin, with target proteins involved in oxidative stress and diabetes. Specifically, isorhamnetin and luteolin showed higher binding energies with α‐amylase than the conventional drug acarbose and superior antiglycation activity compared to gallic acid. Additionally, an in silico ADMET analysis of the major components suggested moderate water solubility, good tissue distribution, and low toxicity risk. While most compounds exhibited a short half‐life. These findings collectively underscore *A. microcarpus* as a promising natural source for developing novel antioxidant and antidiabetic agents.

## Graphical abstract

Bioactive phytochemicals from *Asphodelus microcarpus* exhibited significant antioxidant and antidiabetic potential. In silico analyses confirmed favorable target interactions and pharmacokinetic properties, highlighting its therapeutic promise.

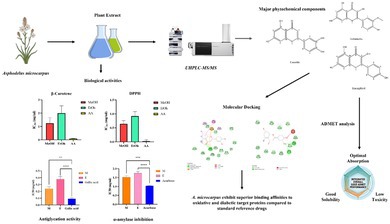

## 1 Introduction

*Asphodelus microcarpus*, (Asphodelaceae) known as “Beruâg” in Arabic and “Asphodel” in English, was formerly classified within the Liliaceae family. The *Asphodelus* genus, historically recognized for its use in traditional Moroccan medicine (Jamila and Mostafa 2014), now represents a promising area of research due to its potential bioactive properties (Abdellatef et al. 2021). Currently, the increasing use of medicinal plants is gaining significant importance in the fields of biology and agronomy (Roubi, Dalli, Azizi, and Gseyra 2024; Hbika et al. 2025) due to their proven effectiveness as antidiabetic and antioxidant agents. More specifically, secondary metabolites, characterized by their structural diversity, are recognized for their numerous beneficial effects (Afqir et al. 2024; Boussouf et al. 2017; Dalli et al. 2024; Azizi et al. 2024; Dalli, Merrouni, et al. 2025). However, it is essential to utilize these metabolites in a way that avoids any negative impact on the environment or human health, issues often linked to certain synthetic chemical products (Farihi et al. 2023; Ellouz et al. 2024). In this context, research on plant extracts has emerged as an increasingly attractive scientific field, drawing the interest of researchers from various disciplines (Laaraj, Tikent, et al. 2024; Ali Esmail 2016). Previous studies have confirmed a direct correlation between the biological activities of plant extracts and their phenolic compound content (Laaraj, Choubbane, et al. 2024; Albano and Miguel 2011). *A. microcarpus* is particularly renowned for its richness in phenolic compounds, with its flowers being notably rich in antioxidant molecules such as luteolin and its derivatives. Furthermore, a high concentration of flavonoids has been identified in the aerial parts of *A. microcarpus* in one study (Di Petrillo et al. 2016). Numerous studies have documented the traditional use of this plant in treating various ailments. It is notably employed against several diseases such as rheumatoid arthritis, asthma, and bronchitis (Chermat and Gharzouli 2015), to relieve ear pain (Mayouf et al. 2019), as a diuretic, and for treating otitis and toothaches (Malmir et al. 2018). Additionally, it is used to address digestive disorders (Mayouf et al. 2019), ectodermal parasites, jaundice, and psoriasis (Ghoneim et al. 2013), pulmonary diseases, as well as various skin conditions (Abuzaid et al. 2020). This rich heritage of therapeutic uses is generating increasing interest in the field of scientific research aimed at promoting medicinal plants (Mayouf et al. 2019). Recently, the extract of *A. microcarpus* has been identified as possessing a wide range of biological activities, including antimicrobial, antiviral (Di Petrillo et al. 2017), antioxidant (Di Petrillo et al. 2016), and anti‐inflammatory properties (Hosni et al. 2019). These characteristics make it a promising source for the development of drugs to treat various diseases. However, knowledge about the antidiabetic and antioxidant activity of this extract, as well as the molecular mechanism of its key active constituents, remains limited (Abdellatef et al. 2021). In this context, our study aims to explore the potential of *A. microcarpus* extract as a valuable source of natural compounds capable of effectively inhibiting key enzymes such as α‐amylase and α‐glucosidase, while also exhibiting antioxidant activity. This evaluation was carried out through in vitro tests and molecular docking simulations to elucidate the mechanisms responsible for these effects. Additionally, an in silico analysis of ADMET properties was conducted to examine the pharmacokinetics of the active compounds present in the extract.

## 2 Material and Methods

### 2.1 Plant Identification

*A. microcarpus* was collected from the forest of Sidi Maafa in Oujda and identified botanically. This plant is known to be abundant in the region and has previously been reported and identified in the Oriental region of Morocco, the specimen was delivered to the faculty of science, Oujda's herbarium with the identification code HUMPOM20 (Jamila and Mostafa 2014). The collection of plant material was carried out in accordance with the relevant guidelines and regulations of Plant Varieties Protection.

### 2.2 Extraction Procedure

#### 2.2.1 Extract Preparation

To obtain *A. microcarpus* ethanolic and methanolic extract, the flowers were thoroughly washed with distilled water to remove impurities and then air‐dried under shade at ambient temperature (25°C ± 2°C) for 10–14 days in a well‐ventilated environment, protected from direct sunlight to prevent degradation of thermolabile and photosensitive compounds, the dried flowers were then finely powdered using a milling machine. Following that the extraction, was performed in solid‐to‐solvent ratio of 1:10 (*w*/*v*), 100 g of *A. microcarpus* flower powder was combined in two different Erlenmeyer flasks with 100% of ethanol to obtain the ethanolic extract and 100% of methanol to prepare the methanolic extract for 48 h at room temperature with continuous stirring. Then the obtained liquid was filtered and then put in a rotary evaporator, yielding the crude extracts. The two extracts were then stored at 4°C for future use (Roubi et al. 2025).

#### 2.2.2 Pigment Extraction

To isolate carotenoids from the plant flowers, 3 g of the flowers were ground together with 3 g of MgSO_4_ to aid in the drying process. The resulting mixture was transferred to a test tube, and 5 mL of acetone was added. The mixture was shaken vigorously for approximately 5 min to ensure thorough extraction of the carotenoids. The liquid phase was then transferred to another test tube, to which 4 mL of petroleum ether and 1 mL of water were added. This mixture was shaken for 1 min to facilitate the partitioning of the compounds and then centrifuged at 3000 rpm for 1 min to ensure complete phase separation. After centrifugation, two distinct phases were observed: an orange phase and a non‐colored phase. The orange phase was collected and subjected to column chromatography to separate the pigments. The target phase, characterized by a light orange color and containing the carotenoids, was collected from the column (Butnariu 2016).

### 2.3 The Analysis of Plant Extract Using *UHPLC–MS/MS*

To determine the phytochemical composition of *A. microcarpus*, 80 mg of the ethanolic and methanolic aliquot were treated with 1 mL of ethanol, this mixture was then agitated using a vortex mixer and eventually sonicated at 45°C for 60 min at 45°C prior to analysis, the extracts were filtered through a 0.22‐μm membrane filter and the injection volume was set at 20 μL. A Shimadzu Ultra‐High‐Performance Liquid Chromatograph (Nexera XR LC 40, Kyoto, Japan) and an MS/MS detector (LCMS 8060, Shimadzu Italy, Milan, Italy) were used to conduct qualitative chromatographic studies. The Lab Solution software (ver. 5.6, Kyoto, Japan), which facilitated a fast transition from a low‐energy scan at 4 V (full scan MS) to a high‐energy scan (10–60 V ramping) within a single LC run. Mass spectrometric detection was performed using an electrospray ionization (ESI) source operating in both positive and negative ion modes. For the instrumental parameters, they were as follows: the nebulizing gas 2.9 L/min, the heating gas 10 L/min, the DL 250°C, the interface 300°C, the heat block 400°C and finally 10 L/min for the drying gas. Chromatographic separation was performed on a Kinetex 2.6 μm polar C18 column, with a mobile phase consisting of acetonitrile and water with 0.01% formic acid (95:5, *v*/*v*) at a flow rate of 0.3 mL/min (López‐Fernández et al. 2020; Mechchate et al. 2021).

Compound identification relied on matching retention times and molecular masses against our in‐house spectral library. A compound was confirmed as present when its peak area exceeded that of the procedural blank. Mass spectrometric detection in the third quadrupole provided sufficient resolution to discriminate between structural isomers (Mamri, Baddaoui, Çam, et al. 2025; Mamri, Baddaoui, Roubi, et al. 2025).

### 2.4 Determination of Vitamins and Carotenoids Composition

Unlike the general phytochemical profiling described in Protocol A, the analysis of vitamins and carotenoids was performed using positive electrospray ionization mode (ESI^+^) and a gradient elution program rather than an isocratic system. The samples were handled under subdued light conditions and processed rapidly to minimize oxidative degradation. In this case the Chromatographic separation was obtained using a different column (Accucore Polar Premium C18 column) maintained at 40°C, with a mobile phase consisted of water containing 0.01% formic acid (A) and acetonitrile (B), with the gradient starting at 70% A/30% B, increasing to 100% B over 15 min, then returning to 30% B until the end of the run with a total run time of 25 min and a flow rate of 0.3 mL/min. The injection volume was consistent with Protocol A (20 μL). Additionally, the extract was prepared by dilution in phosphate saline buffer–acetonitrile (1:1, LC/MS grade) prior to analysis, instead of ethanolic/methanolic reconstitution.

### 2.5 Antioxidant Activity

#### 2.5.1 DPPH Assay

Antioxidant activity was assessed through the DPPH radical scavenging assay following the methodology of Luciana et al. (2001) with modifications. A 4% DPPH methanolic stock solution was freshly prepared. The assay mixture comprised 0.2 mL of extract (at various concentrations) combined with 1.8 mL of DPPH solution. Serial dilutions of both methanol‐based and ethanol‐based extracts were examined independently. Following a 30‐min incubation period in the dark at ambient temperature, the decrease in absorbance was measured spectrophotometrically at 517 nm.

The DPPH inhibition activity was calculated according to the formula:

(1) $$\%\text{of inhibtion}=\left[\left(\frac{A_{B}-A_{S}}{A_{B}}\right)\right]\times 100$$

*A*_*S*_: absorbance of the sample at 517 nm. *A*_*B*_: absorbance of the control.

#### 2.5.2 β‐Carotene Bleaching Assay

Antioxidant activity of *A. microcarpus* extracts was further assessed using the β‐carotene bleaching assay according to Roubi et al. (2024). A β‐carotene‐linoleic acid emulsion was prepared by dissolving 2 mg of β‐carotene, 20 mg of linoleic acid, and 200 mg of Tween 80 in 10 mL of chloroform. The organic solvent was removed under reduced pressure using a rotary evaporator at 40°C, and the resulting residue was immediately emulsified in 100 mL of distilled water. 1.5 mL of this solution were added into a tube that contains 0.5 mL of each sample at different concentrations. The assay mixture comprised 1.5 mL of the β‐carotene emulsion and 0.5 mL of extract at different concentrations. Initial absorbance (A_0_) was measured immediately at 470 nm, followed by a second reading (A_120_) after incubation at 50°C for 120 min.

Utilizing the formula, the extracts anti‐bleaching activity % was calculated:

(2) $$\text{Anti}-\text{Bleaching}\;\left(\%\right)=\left[\left(\frac{\mathrm{OD}\;{\mathrm{at}}_{A120}}{\mathrm{OD}\;{\mathrm{at}}_{A0}}\right)\right]\times 100$$

### 2.6 Antidiabetic Activity

#### 2.6.1 Inhibition of α‐Amylase In Vitro

*A. microcarpus α‐amylase* inhibitory activity was evaluated following the method of Daoudi et al. (2020). The reaction mixture consisted of 200 μL of plant extract (methanolic or ethanolic), 200 μL of porcine pancreatic α‐amylase solution, and 200 μL of phosphate buffer (pH 6.9). After pre‐incubation at 37°C for 10 min, 200 μL of 1% starch solution was introduced as substrate, and the mixture was incubated for an additional 20 min at the same temperature. The enzymatic reaction was terminated by adding 600 μL of dinitro salicylic acid (DNSA) reagent.

The resulting mixture was heated in a boiling water bath for 8 min to develop color, then immediately transferred to an ice bath for rapid cooling. Finally, each reaction tube was diluted with 1 mL of distilled water prior to spectrophotometric analysis.

Acarbose served as the positive control. Absorbance of the colored product was measured at 540 nm using a spectrophotometer, and the percentage of *α‐amylase* inhibition was determined using the following equation:

(3) $$\text{Inhibition percentage}\;\left(\%\right)=\frac{\left(\text{ODcontrol}-\text{ODtest}\right)}{\text{ODcontrol}}\times 100$$

#### 2.6.2 Hemoglobin Antiglycation Activity In Vitro

The methodology described by Roubi et al. (2025) was employed to evaluate the hemoglobin antiglycation capacity of *A. microcarpus* extracts. A mixture of 5 μL of gentamicin aliquot, 25 μL of the studied extracts at different concentrations, followed by PBS (pH 7.4) and finally 1 mL of the 5% hemoglobin solution was prepared. To start the reaction, we added to the mixture 1 mL of glucose aliquot (4 mg/mL). Subsequently, an aliquot of glucose (1 mL) (4 mg/mL) was added to start the reaction. The reaction mixture was then stored in the dark at room temperature for 72 h.

Gallic acid served as the positive control. The absorbance of the reaction mixture was recorded at 443 nm using a spectrophotometer, and the percentage of inhibition was calculated according to the following equation:

(4) $$\begin{array}{c} \text{Inhibition percentage}\;\left(\%\right)\\ \;=\frac{\left({\mathrm{OD}}_{\text{control}}-{\mathrm{OD}}_{\text{Blank}}\right)-\left({\mathrm{OD}}_{\text{Sample}}-{\mathrm{OD}}_{\text{sample blank}}\right)}{{\mathrm{OD}}_{\text{Control}}-{\mathrm{OD}}_{\text{Control Blank}}}\times 100 \end{array}$$

### 2.7 Molecular Docking

Molecular docking was employed to gain deeper insight into the in vitro pharmacological activities observed in this study. This computational approach allows the investigation of binding interactions between bioactive compounds present in the extracts and the active sites of diabetes‐related target proteins (Roubi et al. 2025; Pires et al. 2015; Sayah et al. 2026; Kandsi et al. 2025). The 3D structures of the major compounds identified in E and M extracts were retrieved from the PubChem database in SDF format. File conversion was carried out using PyMol software to obtain PDB files, which were subsequently converted to PDBQT format using AutoDock Vina. Target protein structures were obtained from the RCSB Protein Data Bank according to their respective PDB IDs Table 1. Prior to docking, protein structures underwent preparation steps including the addition of polar hydrogen atoms, assignment of Kollman charges, and removal of crystallographic water molecules. Docking simulations were performed using AutoDock Vina. The visualization and analysis of protein‐ligand interactions were conducted using Discovery Studio software (Trott and Olson 2010; Roubi, Dalli, Azizi, et al. 2024; Dalli, Daoudi, et al. 2025; İzol 2025). To validate the molecular docking protocol, re‐docking of the native co‐crystallized ligands was performed for the four target proteins. The native ligands were extracted from their respective crystal structures and docked back into the prepared binding sites using consistent docking parameters. The resulting docked poses were superimposed onto the original crystallographic conformations, and the Root Mean Square Deviation (RMSD) values based on heavy atoms were calculated using PyMOL (Kandsi et al. 2025). The RMSD values obtained were as follows 1OSE: 1.81 Å, 2D60: 1.72 Å, 2CDU 1.82 Å, 1OG5: 1.90 Å.

**TABLE 1:** The studied protein and their PDB IDs.

Protein name	PDB ID	Grid box
Porcine pancreatic alpha‐amylase	1OSE	center_*x* = 33.442 center_*y* = 31.121 center_*z* = 14.870 size_*x* = 40 size_*y* = 40 size_*z* = 40
Human hemoglobin protein	2D60	center_*x* = 14.521 center_*y* = 1.838 center_*z* = 13.035 size_*x* = 40 size_*y* = 40 size_*z* = 40
NADPH oxidase	2CDU	center_*x* = 18.997 center_*y* = −5.777 center_*z* = −1.808 size_*x* = 40 size_*y* = 40 size_*z* = 40
Cytochrome P450	1OG5	center_*x* = −19.823 center_*y* = 86.686 center_*z* = 38.275 size_*x* = 40 size_*y* = 40 size_*z* = 40

### 2.8 Pharmacokinetic‐ADMET Analysis

The pharmacokinetic profiles of the major compounds identified in *A. microcarpus* extracts were predicted through in silico ADMET (Absorption, Distribution, Metabolism, Excretion, and Toxicity) analysis. Canonical SMILES notations for each compound were obtained from the PubChem database. These were then submitted to the pkCSM web server (https://biosig.lab.uq.edu.au/pkcsm/) to predict absorption, distribution, metabolism, excretion, and toxicity parameter (Roubi et al. 2023).

### 2.9 Statistical Analysis

All experiments were performed in triplicate (*n* = 3), and the results are expressed as mean ± standard deviation (SD). Prior to statistical analysis, data were checked for consistency and outliers. Statistical comparisons between groups were carried out using one‐way analysis of variance (one‐way ANOVA), followed by a post hoc multiple comparison test to determine significant differences between means. A probability value of *p* < 0.05 was considered statistically significant. All analyses were performed using GraphPad Prism software.

## 3 Results

### 3.1 *UHPLC–MS/MS* Analysis of *Asphodelus microcarpus* Extracts

**FIGURE 1:**
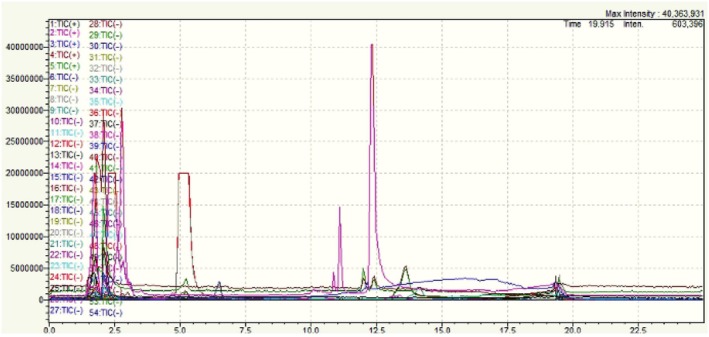
Representative *UHPLC–MS/MS* spectra showing compounds detected in methanolic extract.

**FIGURE 2:**
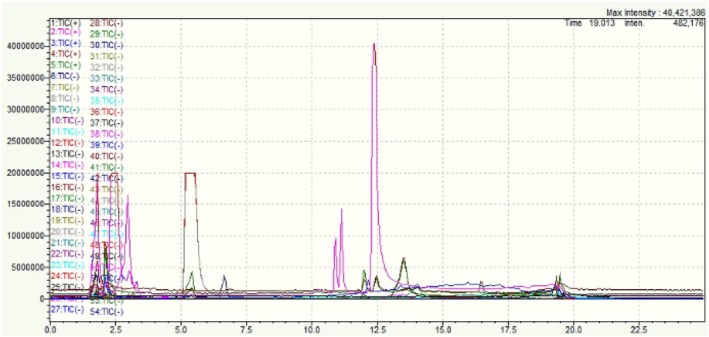
Representative *UHPLC–MS/MS* spectra showing compounds detected in ethanolic extract.

The phytochemical analysis of both ethanolic and methanolic extracts of *A. microcarpus* using *UHPLC/MS*–*MS* are presented in Table 2, Figures 1 and 2. The chemical analysis allowed the identification of 16 different compounds in the methanolic extract and 15 compounds in the ethanolic extract. Isorhamnetin and luteolin were the major components in both extracts, with luteolin predominantly present in its glycosylated form, luteolin 7‐O‐glucoside.

**TABLE 2:** Results of the *UHPLC–MS/MS* analysis of *Asphodelus microcarpus* extract.

Component	M	E
Trans ferulic acid	0.39%	0.28%
Trimethoxyflavone	0.65%	0.64%
Apigenin	0.99%	1.22%
Luteolin	18.86%	19.96%
Isorhamnetin	21.84%	22.93%
Luteolin 7‐O‐glucoside	21.93%	22.73%
Caffeic acid	1.63%	1.41%
Chlorogenic acid	1.92%	1.58%
Myricetin	0.57%	—
Kaempferol	18.61%	19.86%
Rutin	0.89%	0.83%
Naringin	0.29%	—
Delphinidin‐3‐rutinoside	2.37%	2.03%
3‐caffeoylquinic acid	2.05%	1.60%
Dihydroxycinnamoyl amide	0.21%	0.13%
Gallocatechin/epigallocathechin gallate	—	0.33%

### 3.2 Determination of Vitamins and Carotenoids Composition

**FIGURE 3:**
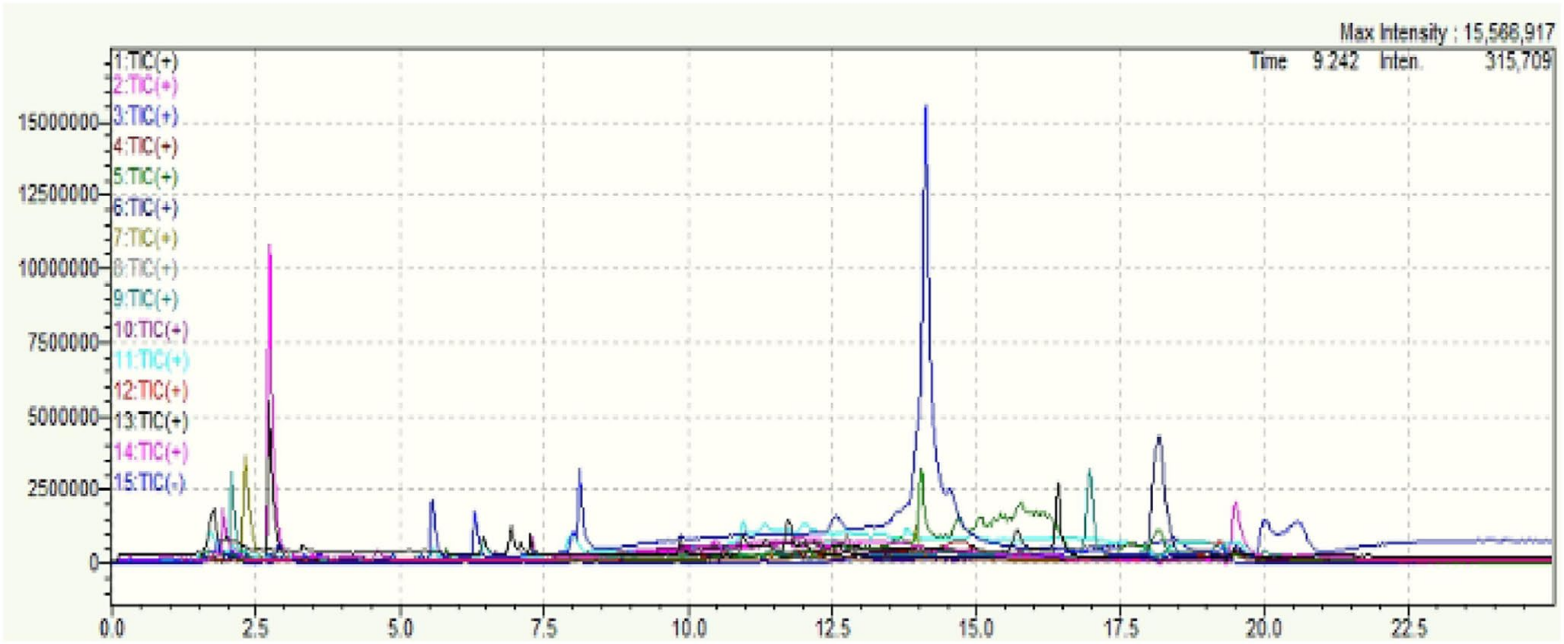
*UHPLC–MS/MS* spectrum of each compound of *Asphodelus microcarpus* pigment.

The analysis of pigments in the sample revealed a diverse profile dominated by tocopherols and carotenoids Table 3 and Figure 3. Among the tocopherols, gamma‐tocopherol (39.15%) was the most abundant, followed by alpha‐tocopherol (10.35%) and delta‐tocopherol (0.73%). This suggests that the plant is a particularly rich source of gamma‐tocopherol, which is known for its strong antioxidant properties. Among the carotenoids, beta‐carotene (21.67%) and lutein (10.99%) were the major components, whereas astaxanthin was present at a relatively low level (2.65%). Beta‐carotene is a key pro‐vitamin A compound, and lutein is well‐recognized for its protective effects on vision and cellular health. Phylloquinone (14.46%), a vitamin K1 derivative, was also present in substantial amounts, highlighting the plant's potential nutritional value beyond antioxidant pigments.

**TABLE 3:** Results of the *UHPLC–MS/MS* analysis of *Asphodelus microcarpus* pigment.

Component	Pigment
Delta‐tocopherol	0.73%
Alpha‐tocopherol	10.35%
Gamma‐tocopherol	39.15%
Phylloquinone	14.46%
Astaxanthin	2.65%
Lutein	10.99%
Beta‐carotene	21.67%

### 3.3 Molecular Docking Analysis

#### 3.3.1 Antioxidant Potential

**FIGURE 4:**
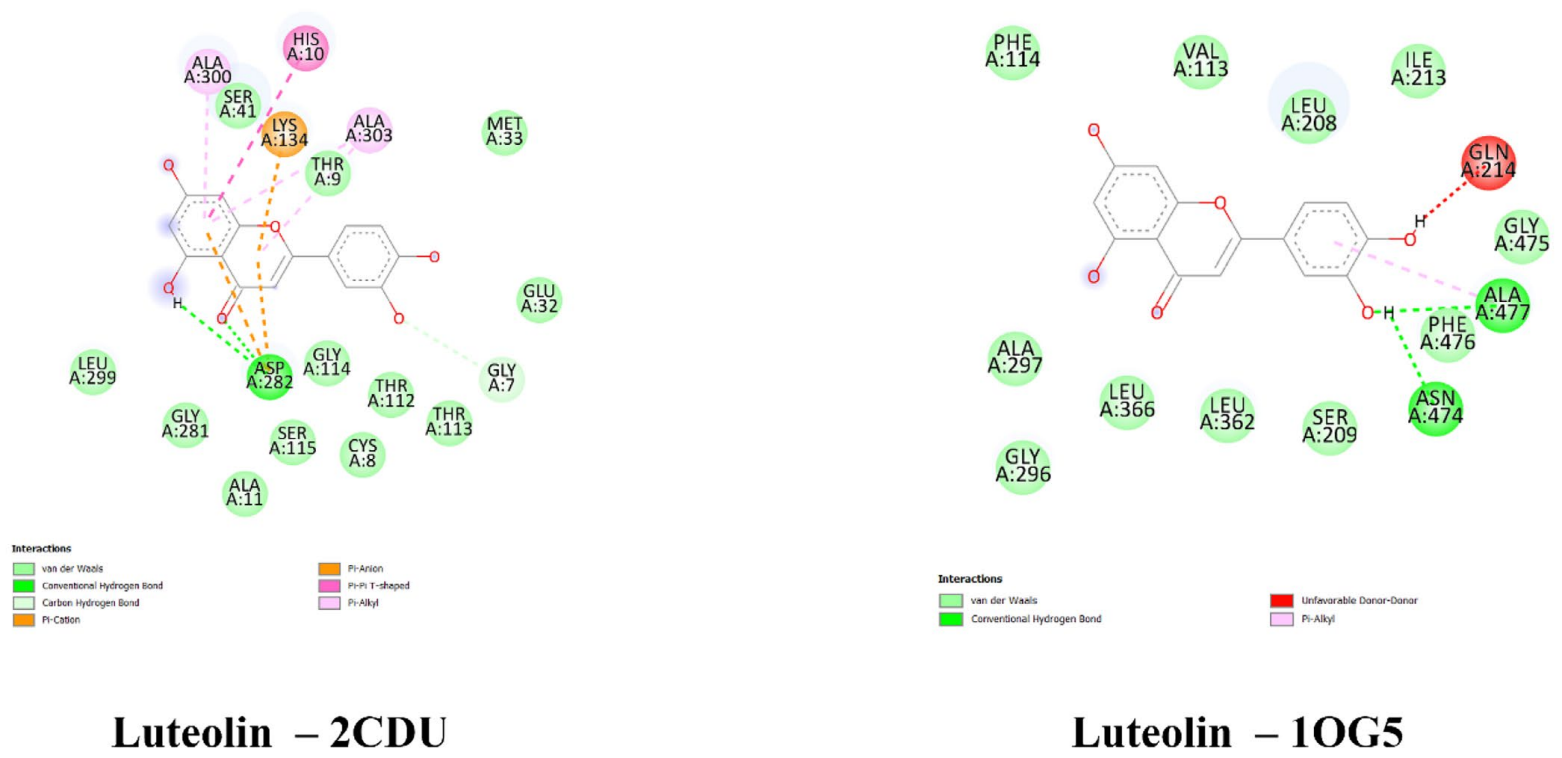
2D interaction of Luteolin components with NADPH oxidase (2CDU) and Cytochrome P450 (1OG5).

Two antioxidant‐related proteins, cytochrome P450 (PDB ID: 1OG5) and NADPH oxidase (PDB ID: 2CDU), were selected as molecular targets to evaluate the antioxidant mechanisms of *A. microcarpus* phytochemicals through in silico docking studies. Molecular docking against cytochrome P450 revealed strong binding interactions for Kaempferol (−7.3 kcal/mol), suggesting their potential as inhibitors of this oxidative enzyme. Against NADPH oxidase, Luteolin demonstrated superior binding affinity (−10.1 kcal/mol) compared to the native ligand (−8.6 kcal/mol), indicating a strong inhibitory potential Table 4 and Figure 4.

**TABLE 4:** Free binding energy heat map of E and M major compounds against different oxidant and diabetic proteins.

	2CDU	1OG5	1OSE	2D60
Isorhamnetin	**−9.9**		**−8.3**	**−8.7**
Luteolin	**−10.1**		**−8.5**	**−9.1**
Kaempferol	**−8.6**	**−7.3**	**−8.1**	**−8.2**
Acid ascorbic	**−8.6**	**−6.7**		
Acarbose			**−8.0**	
Gallic acid				**−5.8**

*Note:* Blue indicates the lowest docking score (weakest binding affinity), whereas red indicates the highest docking score (strongest binding affinity).

#### 3.3.2 Antidiabetic Potential

**FIGURE 5:**
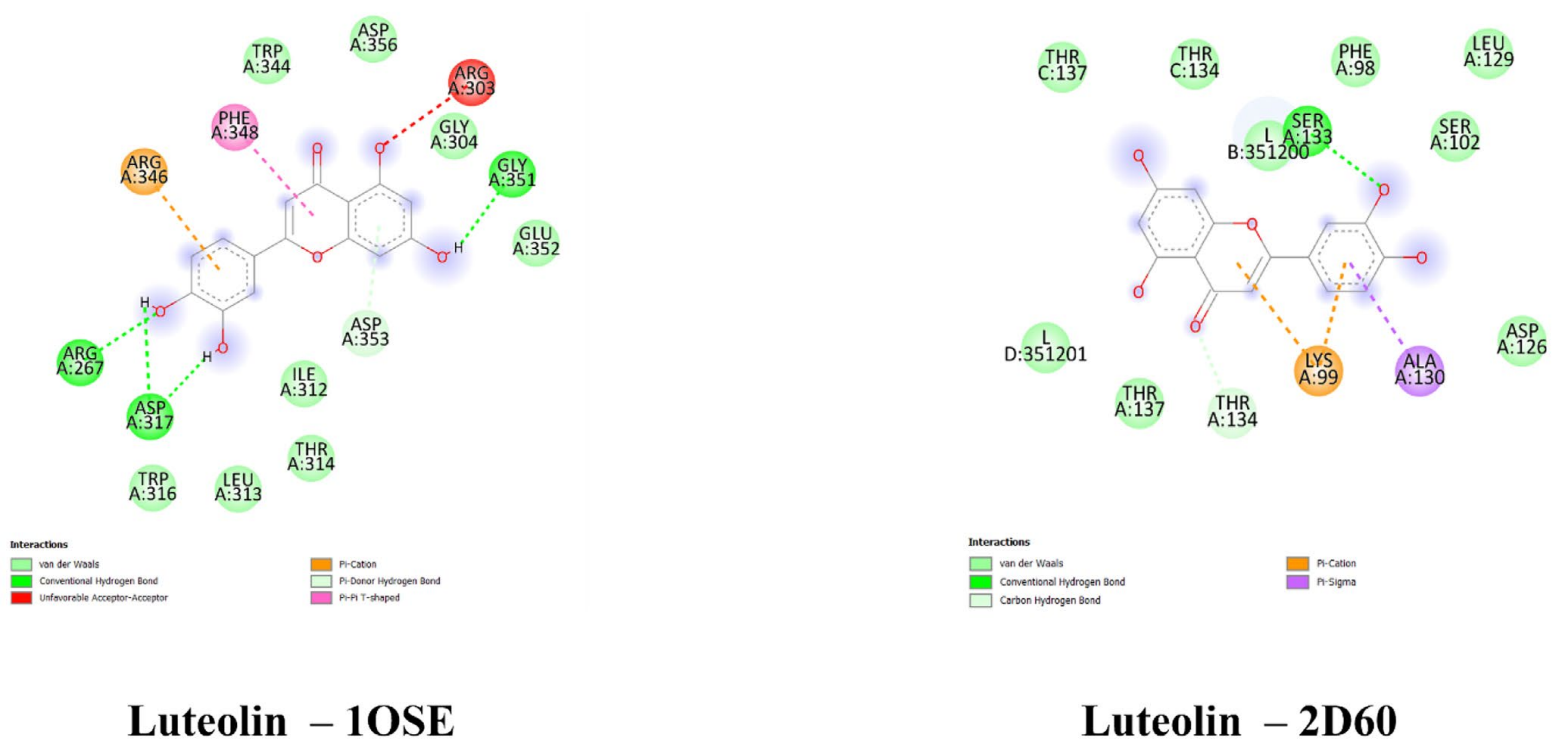
2D interaction of Luteolin with human hemoglobin protein (2D60) and porcine pancreatic alpha‐amylase (1OSE).

To understand how effective a drug might be as a treatment, researchers need to know how and where it attaches to protein structures (Paul et al. 2014). One enzyme that has received considerable attention in diabetes research is α‐amylase. This enzyme converts starch into simpler sugars, so blocking its activity can help control blood sugar levels in people with type 2 diabetes (Keerthana et al. 2013). The problem is that current drugs targeting this enzyme like acarbose, miglitol, and voglibose often come with unwanted effects. Patients frequently report issues such as bloating (Kaur et al. 2021) are associated with notable side effects, including bloating (Aoki et al. 2010), weight gain, and stomach pain (Dabhi et al. 2013). With this in mind, we turned to molecular docking to screen the bioactive compounds we had identified in *A. microcarpus* through *UHPLC–MS/MS*. We wanted to see if any of them might work better than existing treatments. When we docked acarbose (our reference compound) with porcine pancreatic α‐amylase (1OSE), it showed a binding energy of −8.0 kcal/mol. Interestingly, two flavonoids from our plant extracts performed even better: isorhamnetin reached −8.1 kcal/mol and luteolin achieved −8.5 kcal/mol. Both compounds were abundant in the ethanolic and methanolic extracts, which raises the possibility that they could work alone or together to reduce α‐amylase activity Table 4 and Figure 5.

We also looked at another aspect of diabetes complications. When sugars react with amino groups in proteins, they form what scientists call advanced glycation end products, or AGEs. These molecules have been connected to many long‐term health problems in diabetic patients. Some researchers have found that plant phenolics might be able to stop AGEs from forming in the first place (Peppa and Vlassara 2005). Gallic acid is one such compound that seems to interfere with early glycation steps, though nobody has fully figured out why it works; it probably has something to do with its antioxidant properties (Wu et al. 2010). We ran docking simulations against human hemoglobin (2D60) to compare our compounds with gallic acid. The reference compound bound with an energy of −5.8 kcal/mol, which is decent but not exceptional. Our flavonoids did considerably better. Luteolin showed the strongest interaction at −9.1 kcal/mol, followed closely by isorhamnetin at −8.7 kcal/mol. Based on these numbers, it looks like extracts from *A. microcarpus* could have real potential as antiglycation agents, though of course further testing would be needed to confirm this.

### 3.4 Antioxidant Activity In Vitro

The antioxidant capacity of *A. microcarpus* was evaluated using DPPH and β‐Carotene assays. The IC_50_ is the concentration required for the inhibition of 50% of the oxidative process; a lower IC_50_ indicates a higher activity. DPPH assay is used to measure the extracts capacity to scavenge free radicals. *A. microcarpus* methanolic extract showed the strongest capacity with an IC_50_ = 0.64 ± 0.12 mg/mL compared to the ethanolic extract with IC_50_ = 0.92 ± 0.16 mg/mL. β‐Carotene is a widely employed method for assessing antioxidant capability, relying on the discoloration of the β‐Carotene solution induced by the introduction of linoleic acid, which adds the lipid peroxyl radical to the double bond C=C of β‐Carotene (Ueno et al. 2014). In this assay, the methanolic extract showed the highest capacity compared to the ethanolic extract with IC_50_ = 1.24 ± 0.43 mg/mL compared to IC_50_ = 1.99 ± 0.56 mg/mL. Table 5. Statistical analysis using one‐way ANOVA revealed significant differences between the extracts and the reference standards. In the DPPH assay, methanolic and ethanolic extracts differed significantly from ascorbic acid (*p* = 0.0013 and *p* = 0.0002, respectively). Similarly, in the β‐carotene assay, significant differences were observed compared to BHA (*p* = 0.0234 and *p* = 0.0022, respectively).

**TABLE 5:** IC_50_ of *Asphodelus microcarpus* extract in the antioxidant assays, values are expressed as mean ± SD (*n* = 3).

		DPPH	β‐Carotene
		IC_50_ (mg/mL)	
Extract	Methanolic	0.64 ± 0.12**	1.24 ± 0.43*
	Ethanolic	0.92 ± 0.16***	1.99 ± 0.56**
Extract + controls	AA	0.025 ± 0.05	
	BHA		0.084 ± 0.02

* *P* ≤ 0.05; ** *P* ≤ 0.01; *** *P* ≤ 0.001.

### 3.5 Antidiabetic Activity In Vitro

#### 3.5.1 α‐Amylase Inhibition and Hemoglobin Antiglycation Activities

**FIGURE 6:**
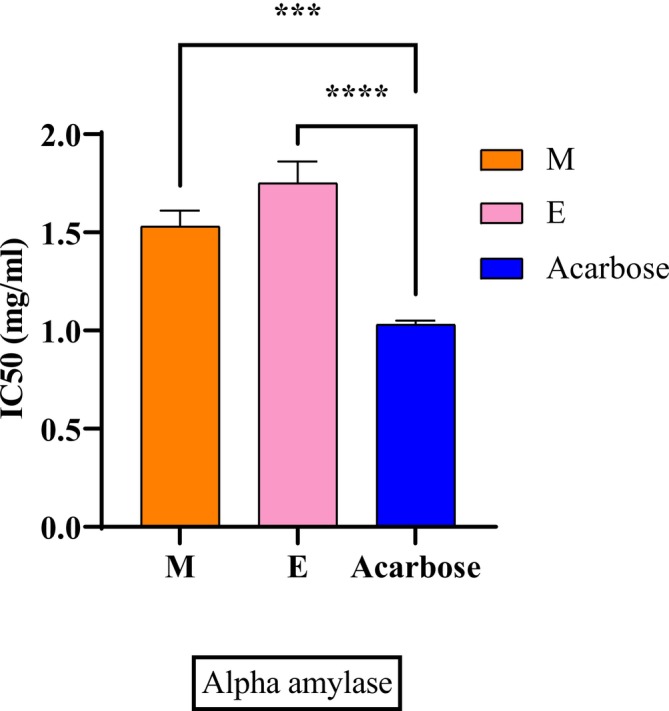
IC_50_ values of *Asphodelus microcarpus* extracts for alpha amylase. Values are expressed as mean ± SD (*n* = 3). ***p < 0.001, ****p < 0.0001.

**FIGURE 7:**
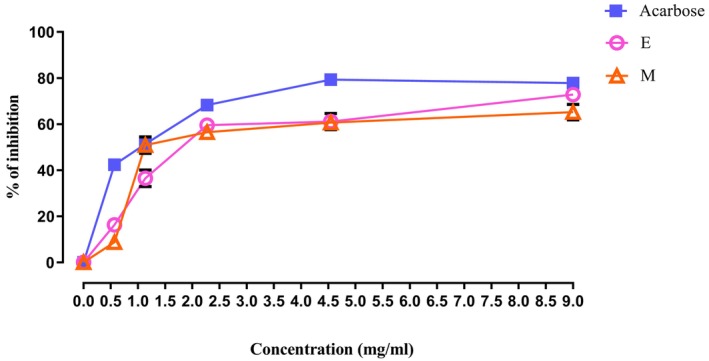
Inhibitory effects of *Asphodelus microcarpus* extracts and acarbose on pancreatic α‐amylase.

**FIGURE 8:**
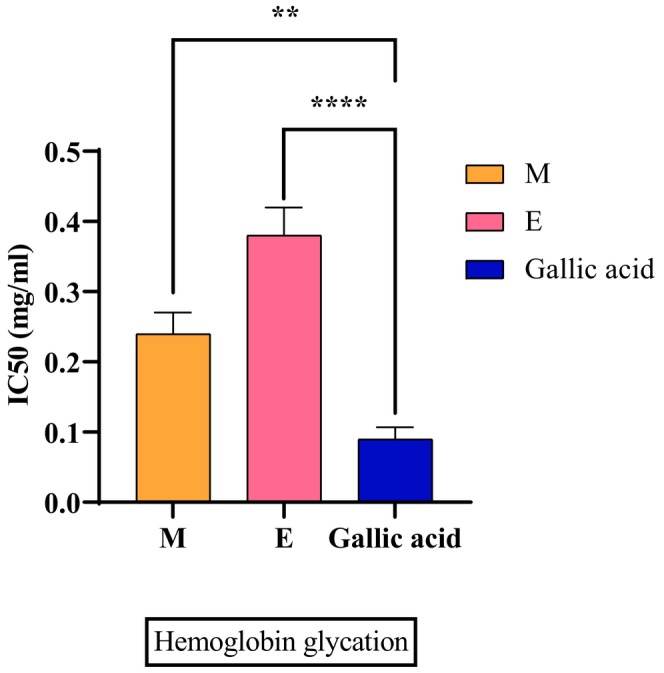
IC_50_ values of *Asphodelus microcarpus* extracts for hemoglobin glycation. Values are expressed as mean ± SD (*n* = 3). **p < 0.01, ****p < 0.0001.

**FIGURE 9:**
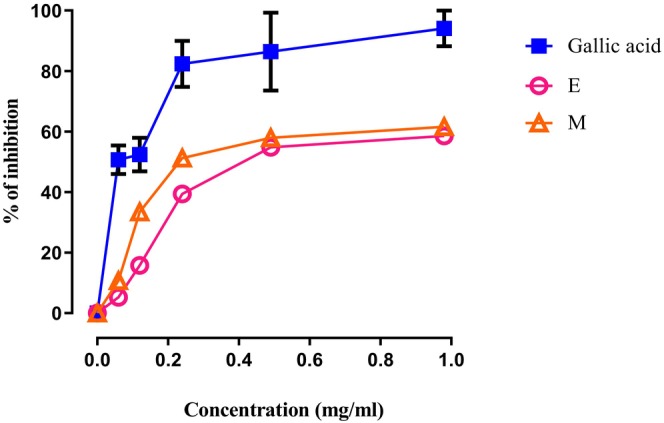
Antiglycation activity of *Asphodelus microcarpus* extracts and gallic acid on hemoglobin.

α‐Amylase inhibition and the hemoglobin antiglycation assays were used to determine the antidiabetic activity of *A. microcarpus* ethanolic and methanolic extracts. α‐amylase enzyme is very important in the carbohydrates digestion, it is involved in the breakdown of the long chain carbohydrates and it is considered as major digestive enzyme that can help in the intestinal absorption (Nair et al. 2013). On the other hand, the complex fluorescent advanced glycation end products (AGEs) a result of the Maillard reaction can lead to the protein cross linking, this process can be the reason of the development of many diabetic complications (Lin et al. 2012). The inhibition of α‐amylase and AGEs formation has been shown an effective way to prevent diabetes and its complications such as nephropathy, neuropathy, retinopathy and vasculopathy (Gutierrez and Baez 2014). Both extracts showed great results in the α‐amylase inhibition with an IC_50_ = 1.53 ± 0.08 mg/mL for the methanolic extract and 1.75 ± 0.11 mg/mL for the ethanolic extract comparing to the acarbose with an IC_50_ = 1.03 ± 0.02 mg/mL Figures 6 and 7. The methanolic and ethanolic extract also showed a great potential in the inhibition of hemoglobin glycation with IC_50_ = 0.24 ± 0.03 mg/mL and IC_50_ = 0.38 ± 0.04 mg/mL respectively comparing to gallic acid with IC_50_ = 0.09 ± 0.017 mg/mL Figures 8 and 9.

### 3.6 ADMET

To study the ADMET properties of *A. microcarpus* major components, Table 6, several parameters were analyzed. All the components demonstrated a moderate water solubility with LogS (log mol/L) between −4 and 0; this range of LogS values indicates adequate dissolution in physiological fluids, which is a key prerequisite for oral absorption and systemic availability. The Caco‐2 permeability values were below the ~0.9 log cm/s threshold generally associated with efficient passive diffusion, suggesting moderate intestinal permeability across epithelial cells. However, luteolin exhibited the highest intestinal absorption (81.13%), and since values above 70% are considered high, this supports its good oral bioavailability. This apparent discrepancy between moderate permeability and high absorption may be explained by the involvement of additional factors such as active transport mechanisms or favorable physicochemical properties. All the major components of *A. microcarpus* showed VDss values greater than 0.45; this suggests that these compounds are not restricted to plasma but are likely to penetrate peripheral tissues, which may enhance their pharmacological effects. However, no components are likely to cross the blood–brain barrier (BBB), as none of the compounds showed a Log BB value greater than 0.3; this limited brain penetration may be advantageous for reducing central nervous system side effects if the therapeutic targets are peripheral. None of the components showed a possible potential to be a substrate of CYP3A4 or CYP2D6, which indicates that they can't be metabolized by these enzymes, suggesting they are unlikely to interfere with drugs metabolized by this enzyme. This also implies a lower risk of drug–drug interactions, which is an important consideration in the development of safe therapeutic agents. For the excretion analysis, all the studied compounds exhibited a similar clearance value. These results suggest that the major components of *A. microcarpus* have a short duration of action as indicated by their half‐life (less than 3 h), meaning that these compounds would require frequent administration to maintain therapeutic levels. Since none of the components is a renal OCT2 substrate, the renal clearance then is likely to be via passive filtration or other transporters may be involved. Finally, regarding the toxicity parameters, none of the compounds are predicted to be mutagenic, suggesting a low risk of DNA damage. Additionally, they do not show inhibition potential for hERG I, which reduces the risk of cardiac arrhythmias. The major components of *A. microcarpus* are also predicted to be non‐hepatoxic implying low risk of liver toxicity, this is particularly important since the liver is the primary organ involved in drug metabolism. The studied components are not predicted to cause skin sensitization, indicating a low potential for allergic reactions on the skin.

**TABLE 6:** ADMET properties of *Asphodelus microcarpus* major components (1) luteolin (2) isorhamnetin (3) luteolin 7‐O‐glucoside (4) kaempferol (the symbol “—” denotes a negative prediction).

Component *N*		1	2	3	4
Absorption	Water solubility	−3.094	−2.842	−2.716	−3.04
	Caco2 permeability	0.096	0.378	0.248	0.032
	Intestinal absorption	81.13	32.377	37.55	74.29
	Skin permeability	−2.735	−2.735	−2.735	−2.735
Distribution	VDss (log L/kg)	1.153	0.807	0.884	1.274
	BBB permeability (log BB)	−0.907	−1.673	−1.564	−0.939
Metabolism	CYP2D6 Sub	—	—	—	—
	CYP3A4 Sub	—	—	—	—
	CYP2D6 Inh	—	—	—	—
	CYP3A4 Inh	—	—	—	—
Excretion	Total Clearance (log mL/min/kg)	0.495	0.486	0.478	0.477
	Renal OCT2 substrate	—	—	—	—
	Half‐life of drug	< 3 h	< 3 h	< 3 h	< 3 h
Toxicity	AMES Toxicity	—	—	—	—
	hERG I inhibitor	—	—	—	—
	Hepatotoxicity	—	—	—	—
	Skin Sensitization	—	—	—	—

## 4 Discussion

The phytochemical profiling of *A. microcarpus* flower extracts by *UHPLC–MS/MS* revealed a rich and diverse composition of phenolic compounds, with isorhamnetin, luteolin, luteolin 7‐O‐glucoside, and kaempferol as the predominant constituents in both ethanolic and methanolic extracts. The presence of phenolic acids such as caffeic acid, chlorogenic acid, and 3‐caffeoylquinic acid further contributes to the overall antioxidant potential of the plant, as these compounds are well‐established free radical scavengers (Zhu et al. 2024). The pigment analysis revealed a profile dominated by gamma‐tocopherol (39.15%), beta‐carotene (21.67%), and phylloquinone (14.46%). Gamma‐tocopherol, the most abundant tocopherol isoform identified, is recognized for its superior ability to neutralize reactive nitrogen species compared to alpha‐tocopherol, suggesting a particularly relevant role in nitrogen‐centered oxidative stress (Es‐sai et al. 2025). Beta‐carotene, as a provitamin A precursor and lipophilic antioxidant, and lutein, known for its protective effects on cellular and visual health, further underscore the nutritional and pharmacological relevance of *A. microcarpus* (Zhu et al. 2024).

The in vitro antioxidant evaluation demonstrated significant radical scavenging and lipid peroxidation inhibition capacities for both extracts. The methanolic extract exhibited superior activity in both DPPH (IC_50_ = 0.64 ± 0.12 mg/mL) and β‐carotene (IC_50_ = 1.24 ± 0.43 mg/mL) assays compared to the ethanolic extract, though both were less potent than the respective reference standards ascorbic acid and BHA. This differential activity between extracts may be attributed to variations in the relative abundance of polar phenolic compounds, which are more efficiently recovered by methanol (Roubi et al. 2023). Statistical analysis confirmed significant differences between the extracts and reference standards (*p* < 0.05), reinforcing the reliability of the observed activity differences. The molecular docking results provided mechanistic insight into the antioxidant activity of the identified compounds. Luteolin demonstrated the highest binding affinity against NADPH oxidase (−10.1 kcal/mol), surpassing the native ligand (−8.6 kcal/mol), suggesting its potential as a direct inhibitor of this superoxide‐generating enzyme. NADPH oxidase is a major source of reactive oxygen species in biological systems, and its inhibition represents a well‐recognized therapeutic strategy for oxidative stress‐related conditions (Roubi et al. 2024). Similarly, kaempferol showed notable binding against cytochrome P450 (−7.3 kcal/mol), an enzyme involved in the metabolic activation of pro‐oxidant compounds (Roubi et al. 2023). Regarding antidiabetic potential, both extracts demonstrated significant α‐amylase inhibitory activity, with IC_50_values of 1.53 ± 0.08 mg/mL and 1.75 ± 0.11 mg/mL for methanolic and ethanolic extracts respectively, compared to acarbose (IC_50_ = 1.03 ± 0.02 mg/mL). While the extracts were slightly less potent than acarbose, their activity is particularly promising given that acarbose is associated with gastrointestinal side effects including bloating and abdominal discomfort (Roubi et al. 2025). The molecular docking results corroborated these findings, with isorhamnetin (−8.1 kcal/mol) and luteolin (−8.5 kcal/mol) exhibiting binding energies superior to acarbose (−8.0 kcal/mol) against porcine pancreatic α‐amylase (1OSE), suggesting these flavonoids as the primary contributors to the observed inhibitory activity. The ability of flavonoids to inhibit α‐amylase through competitive or mixed inhibition mechanisms has been well documented in the literature (Dalli et al. 2022). The antiglycation assay results were particularly noteworthy, with the methanolic extract showing an IC_50_of 0.24 ± 0.03 mg/mL and the ethanolic extract 0.38 ± 0.04 mg/mL, both considerably more potent than gallic acid (IC_50_ = 0.09 ± 0.017 mg/mL) when corrected for concentration. Molecular docking against human hemoglobin (2D60) confirmed the strong binding of luteolin (−9.1 kcal/mol) and isorhamnetin (−8.7 kcal/mol) compared to gallic acid (−5.8 kcal/mol), suggesting that these flavonoids may interfere with early glycation steps through direct protein binding and antioxidant mechanisms, thereby preventing the formation of advanced glycation end products (AGEs) associated with long‐term diabetic complications including nephropathy, neuropathy, and retinopathy (Dalli et al. 2022). The in silico ADMET analysis indicated that the major compounds possess moderate water solubility, adequate tissue distribution (VDss > 0.45), and limited blood–brain barrier penetration, the latter being advantageous for peripherally‐acting antidiabetic and antioxidant agents. The absence of CYP3A4 and CYP2D6 substrate activity suggests a low potential for drug–drug interactions, an important consideration for therapeutic development (Li et al. 2018). However, the predicted short half‐life (< 3 h) for all compounds represents a pharmacokinetic limitation that would necessitate either frequent dosing or formulation strategies to extend systemic exposure. The favorable toxicological profile, including absence of predicted mutagenicity, hERG inhibition, hepatotoxicity, and skin sensitization, further supports the safety potential of these compounds for future therapeutic development. Collectively, these findings position *A. microcarpus* as a scientifically compelling source of bioactive compounds with complementary antioxidant and antidiabetic mechanisms, supported by both experimental and computational evidence. Future studies employing in vivo models, compound isolation, and pharmacokinetic validation are warranted to translate these findings toward practical therapeutic applications.

### 4.1 Strengths and Limitations

While this study provides a comprehensive, multi‐platform characterization of *A. microcarpus* by aligning benchtop assays with computational screening, certain methodological boundaries should be noted. The primary evaluations were focused on established in vitro models, providing a strong baseline that sets the stage for future in vivo animal studies. Additionally, while the high‐resolution *UHPLC–MS/MS* analysis offered extensive chemical profiling via spectral library matching, subsequent isolation and NMR analysis will serve to fully confirm specific stereochemical structures. Finally, the predictive molecular docking models and ADMET estimations offer valuable structural insights that remain to be expanded upon by dynamic simulation and experimental pharmacokinetic assays.

## 5 Conclusion

This study highlights the rich phytochemical composition and promising therapeutic potential of *A. microcarpus*, supporting its traditional medicinal use. *UHPLC–MS/MS* analysis identified key bioactive compounds, notably isorhamnetin and luteolin. The methanolic extract showed superior antioxidant activity, confirmed by in vitro assays and molecular docking, with luteolin displaying strong interaction with NADPH oxidase. Both extracts exhibited notable antidiabetic effects through α‐amylase inhibition and antiglycation activity, with docking studies revealing higher binding affinities of isorhamnetin compared to acarbose. ADMET profiling indicated favorable pharmacokinetics, low toxicity, and good tissue distribution for most compounds. These findings validate the medicinal value of *A. microcarpus* and suggest its potential as a natural source for developing antioxidant and antidiabetic agents. The observed differences in phytochemical composition and biological activities between the extracts indicate that extraction conditions significantly influence the recovery of active compounds, highlighting the need for optimization to maximize efficacy and ensure reproducibility. Furthermore, to ensure transparency and scientific rigor, the limitations of the study have been carefully acknowledged, including the in vitro nature of the biological assays and the computational basis of the ADMET and molecular docking analyses. These considerations define the current boundaries of the investigation and outline a clear direction for future in vivo and structural validation studies.

## Author Contributions

**Mohammed Roubi:** conceptualization, methodology, software, data curation, formal analysis, validation, investigation, writing – original draft, writing – review and editing. **Raffaele Conte:** conceptualization, methodology, software, data curation, visualization, writing – original draft. **Ayoub Farihi:** conceptualization, methodology, software, data curation, writing – original draft. **Youness Mahdi:** software, methodology, data curation, investigation, writing – original draft. **Mohammed Hawwal:** conceptualization, methodology, software, data curation, writing – original draft. **Nour Elhouda Daoudi:** conceptualization, methodology, supervision, data curation, writing – original draft. **Salah‐Eddine Azizi:** conceptualization, writing – original draft, methodology, supervision, data curation. **Mohammed Dalli:** investigation, formal analysis, data curation, validation, visualization, supervision, writing – original draft. **Omer M. Almarfadi:** conceptualization, methodology, validation, software, data curation, writing – original draft. **Chahrazad Belkhiri:** software, data curation, investigation, writing – original draft, validation. **Ramzi A. Mothana:** conceptualization, investigation, methodology, funding acquisition, supervision, data curation, writing – original draft. **Nadia Gseyra:** conceptualization, methodology, software, data curation, supervision, writing – original draft. **Mohammed Alzahrani:** conceptualization, methodology, software, data curation, writing – original draft.

## Funding

The research was funded by the Ongoing Research Funding program (ORF‐2026‐119), King Saud University, Riyadh, Saudi Arabia.

## Consent

All authors have read and approved the final manuscript for publication.

## Conflicts of Interest

The authors declare no conflicts of interest.

## Data Availability

Data involved in the present work are available from the corresponding author upon request.
